# Supplementary material for: CREPE (CREate Primers and Evaluate): A Computational Tool for Large-Scale Primer Design and Specificity Analysis
Source: Genes (Basel). 2025 Sep 10;16(9):1062. doi: 10.3390/genes16091062 (PMC12469620; doi:10.3390/genes16091062)
Supplement: Supplementary file 1 [file genes-16-01062-s001.zip › S_File4_CREPE_README.pdf]

CREPE (CREate Primers and Evaluate)

Overview

CREPE is a batch primer design and specificity analysis tool. It uses Primer3 (https://primer3.org/) to create primers from an input CSV of target sites. It then uses UCSC's In-Silico PCR (https://genome.ucsc.edu/cgi-bin/hgPcr) to identify off-target enrichment sites for each primer pair. Lastly, a custom Python evaluation script (E-script) performs specificity analysis to determine the quality of predicted off-target sites from ISPCR.

The current version of CREPE is designed to create primer pairs for 150bp short-read targeted amplicon sequencing (TAS) applications. As such, it first attempts to find primer pairs that generate a 190bp amplicon that will likely perform well in a TAS experiment. CREPE prioritizes those TAS-optimized (TAS-opt) primer pairs. Through testing, we've found that TAS-opt primer pairs can be made for about 70% of input target sites. For the remaining target sites, CREPE will create alternate primer pairs. This is achieved by giving one of the primers in the reaction a larger area to bind. Relaxed-right primer pairs have the forward primer anchored near the target site, while the reverse primer is given more genomic real estate. Inversely, the Relaxed-left primer pairs have the reverse primer anchored near the target site while the forward primer has a larger region to bind to. Please see the figure below for more detail. The alternate primer amplicons can be as large as 500bp. In this way, CREPE attempts to create functional primer pairs for every input target site.

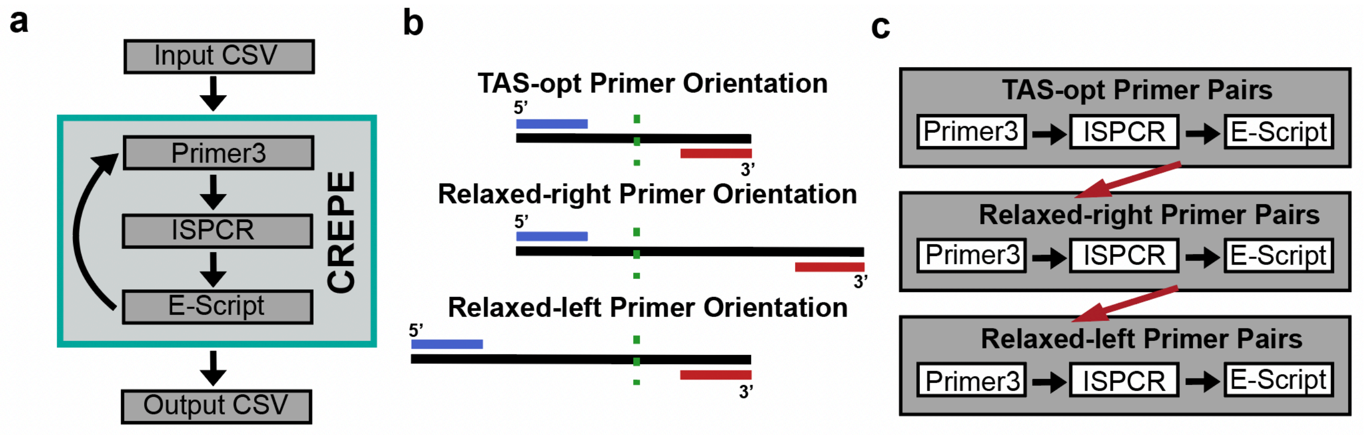

CREPE requires a custom anaconda environment created during installation. It runs efficiently on local machines, although it can be run on an HPC if that's most convenient.

Installation

CREPE runs with few dependencies and a simple conda environment. The suggested installation method is to download and extract the tarball. However, cloning this repository should also work. In any case, the following instructions will help you prepare the files and environment needed for CREPE to run.

1. To begin, download and extract the tar file:

```
tar -xvzf crepe_download.tar.gz
```

2. Build the CREPE anaconda environment by running the following command:

```
conda env create --name crepe --file=env.yaml
```

If you have an issue creating the environment from the env.yaml file, then use the following commands to create your environment:

```
1. conda create -n crepe python=3.9.12
2. conda activate crepe
3. conda install pandas=1.4.2
4. conda install bioconda::pysam
5. conda install conda-forge::biopython=1.79
6. conda install bioconda::bedtools
7. conda install bioconda::primer3
8. conda install bioconda::ispcr
```

It should take less than 10 minutes to build the environment.

3. If you have an issue with the tar file, Git clone this directory:

```
1. git clone -b main https://github.com/martinbreuss/BreussLabPublic
```

Usage

The input CSV file can contain as many columns as you like. However, the chromosome column must be labeled CHROM, the position must be POS, and the project ID must be PROJ. This is shown in the clinvar10_demo.csv file.

1. This is the command structure to run CREPE:

```
python CREPE_v1.02.py input.csv ref_genome.fa output_directory projID
```

2. This is an example command:

```
python CREPE_v1.02.py clinvar10_demo.csv hg38.fa test_crepe test_crepe10
```

The final output file with the list of primers is called: FINAL_OUTPUT_FILE_projID (FINAL_OUTPUT_FILE_test_crepe10). We are working to clean up the output intermediary files. (03/26/2025)

The final output file with all identified off-targets that passed filtering is called: ALL_OFFTARGETS_FOUND_for_projID (ALL_OFFTARGETS_FOUND_for_test_crepe10). Note that this file also contains the on-target primer pair. It's easiest to filter this file to find the off-targets found for specific primers instead of reading the file as a whole.

Below is a sample output from the provided demo file:

CHROM	POS	PROJ	variant_id	primer3	isPcr	primer_name	TAS-opt	forward_nam	forward_prim	forward_tm	reverse_nam	reverse_prim	reverse_tm	amplicon_sta	amplicon_en	amplicon_siz	primer_coun	concerning_off_targets
9	108912753	clinvar	clinvar_9_10	TRUE	TRUE	clinvar_9_10	FALSE	clinvar_9_10	CAGTCCAGA	59.966	clinvar_9_10	GGAGATGGA	59.749	108912479	108912846	367	3	FALSE
7	76066259	clinvar	clinvar_7_76	TRUE	TRUE	clinvar_7_76	TRUE	clinvar_7_76	TAGAGAGAC	59.818	clinvar_7_76	GGATGGCAT	59.446	76066177	76066354	177	1	FALSE
10	43128133	clinvar	clinvar_10_4	TRUE	TRUE	clinvar_10_4	TRUE	clinvar_10_4	CCAAGGCC	59.963	clinvar_10_4	AGCATCCAG	57.185	43128039	43128226	187	1	FALSE
1	201087921	clinvar	clinvar_1_20	TRUE	TRUE	clinvar_1_20	TRUE	clinvar_1_20	CAGCACCA	59.674	clinvar_1_20	CAGGGTTGG	58.642	201087839	201088015	176	1	FALSE
17	61683530	clinvar	clinvar_17_6	TRUE	TRUE	clinvar_17_6	FALSE	clinvar_17_6	TGCAGAATT	57.466	clinvar_17_6	AGAAGCCCT	60.253	61683446	61683758	312	1	FALSE
13	33065695	clinvar	clinvar_13_3	FALSE	FALSE	NA	NA_check_P3	NA	NA	NA	NA	NA	NA	NA	NA	NA	NA	NA
17	29055217	clinvar	clinvar_17_2	TRUE	TRUE	clinvar_17_2	TRUE	clinvar_17_2	GACGTCCA	59.898	clinvar_17_2	CCAGTGCAA	60.321	29055134	29055297	163	1	FALSE
14	38209841	clinvar	clinvar_14_3	TRUE	TRUE	clinvar_14_3	TRUE	clinvar_14_3	CTTCGGTGC	61.485	clinvar_14_3	TTACTACCTT	59.678	38209760	38209921	161	3	TRUE
15	42389022	clinvar	clinvar_15_4	TRUE	TRUE	clinvar_15_4	TRUE	clinvar_15_4	ACGAAGCTC	59.965	clinvar_15_4	CCCACACC	59.008	42388941	42389114	173	1	FALSE
20	63688197	clinvar	clinvar_20_6	TRUE	TRUE	clinvar_20_6	TRUE	clinvar_20_6	CTGAGGTCC	59.404	clinvar_20_6	GCAGGGGC	60.124	63688104	63688274	170	2	FALSE
19	50045372	clinvar	clinvar_19_5	TRUE	TRUE	clinvar_19_5	TRUE	clinvar_19_5	ACCTGTGGC	60.159	clinvar_19_5	TTCTGGTGC	59.68	50045278	50045462	184	2	TRUE

Updates

[redacted]

The Python script will be updated to improve performance and allow users to customize amplicon size for applications other than TAS. Please contact Jon, (jonathan[dot]pitsch@cuanschutz[dot]edu), if you have any issues using CREPE.
