# Supplementary figures and images for: CREPE (CREate Primers and Evaluate): A Computational Tool for Large-Scale Primer Design and Specificity Analysis
Source: Genes (Basel). 2025 Sep 10;16(9):1062. doi: 10.3390/genes16091062 (PMC12469620; doi:10.3390/genes16091062)

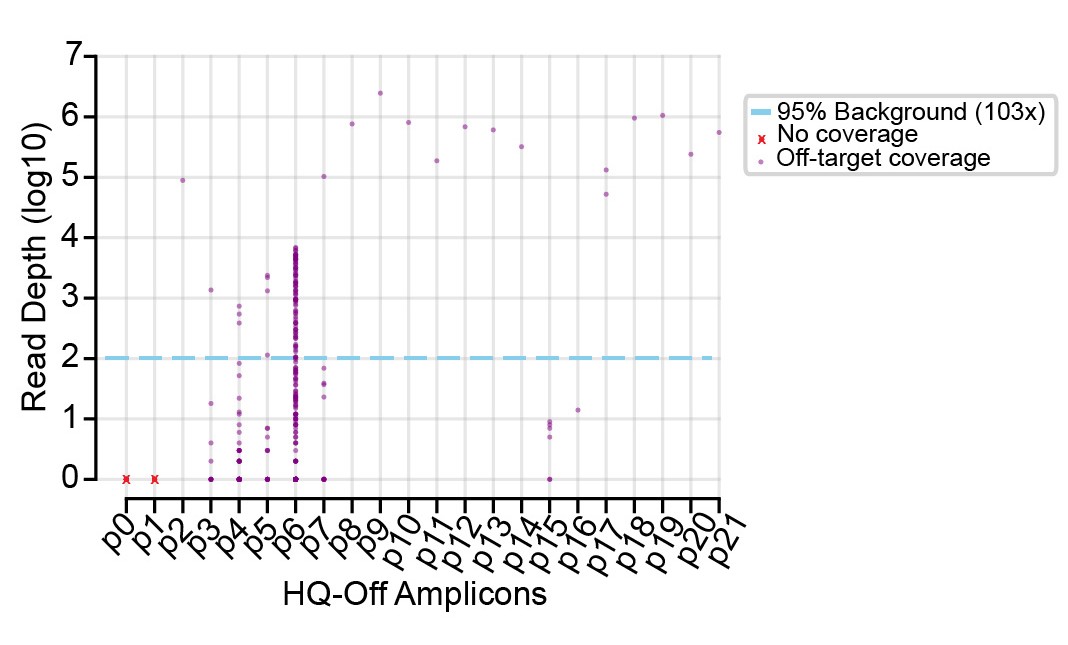

Supplement: Supplementary file 1 [file genes-16-01062-s001.zip › 20250909_FigureS1.jpg]
